# “I think we're on a cusp of some change:” coping and support for mental wellness among Black American women

**DOI:** 10.3389/fpsyg.2024.1469950

**Published:** 2025-01-14

**Authors:** Terika McCall, Meagan Foster, Holly Tomlin, Bolatito Adepoju, Mckenzie Bolton-Johnson, Chyrell D. Bellamy

**Affiliations:** ^1^Division of Health Informatics, Department of Biostatistics, Yale School of Public Health, New Haven, CT, United States; ^2^Department of Biomedical Informatics and Data Science, Yale School of Medicine, New Haven, CT, United States; ^3^Center for Interdisciplinary Research on AIDS (CIRA), Yale School of Public Health, New Haven, CT, United States; ^4^Emory University School of Law, Atlanta, GA, United States; ^5^MMJ Counseling & Consulting, Garner, NC, United States; ^6^Department of Psychiatry, Yale School of Medicine, New Haven, CT, United States

**Keywords:** Black or African American, women, mental health, anxiety, depression, telemedicine, mHealth, digital health

## Abstract

**Introduction:**

Public discussions in the media (e.g., social media and reality shows) about Black women's mental health have become more common. Notably, celebrities have become more vocal about their own mental health challenges and sought to normalize seeking care. This study aimed to gain a better understanding of Black women's: (1) past and current causes of stress, anxiety, and depression, and coping skills used; (2) their attitudes and perceptions toward mental health and receiving mental health treatment; and (3) times in their life they felt anxious or depressed, and what type of support or resources would have been helpful to have access to.

**Methods:**

Focus groups were conducted with 20 women (mean age 36.6 years, SD 17.8 years), with 5 participants per group. Descriptive qualitative content analysis of the focus group transcripts was conducted.

**Results:**

Results consistently showed that intersectional identities of being both Black and a woman resulted in feelings of both hypervisibility and invisibility, representation matters when it comes to mental health providers, an increased openness to therapy across age groups, and a willingness to try digital health tools (e.g., smartphone app) for mental health needs. There is still work to be done to normalize mental health treatment among Black women.

**Discussion:**

Subgroups within the community (e.g., young adults) have less stigma around mental health and are acting as catalysts for change. Intentional inclusion of Black women in mental health research and evolving treatment paradigms is important to eliminate inequities in access to culturally relevant mental health care.

## Introduction

Mental health conditions include a broad range of illnesses and psychosocial disabilities, as well as mental states that can result in substantial distress, functional limitations, or the potential for self-harm (National Institute of Mental Health, 2024). Approximately one in three people have their mental health disturbed at some point throughout their lives (Ginn and Horder, 2012; Steel et al., 2014). Importantly, mood disorders—major depressive disorder in particular—is common among Black women, with an estimated 27% having had depression at some point in their lives (McCall et al., 2023). Black women are particularly impacted by several risk factors, including socioeconomic inequality, prejudice, and increased prevalence of specific illnesses (e.g., type-2 diabetes, obesity, and hypertension) exacerbated by the strain of caregiving, often for multiple generations (Watson and Hunter, 2015). These problems were likely made worse by the COVID-19 pandemic, underscoring the intersectional effects on Black women's mental health of gendered prejudice and stress (McCall et al., 2023; Watson and Hunter, 2015; Okeke, 2013).

In addition to these stressors, Black women encounter obstacles on their way to seeking support and are half as likely to seek mental health treatment in traditional settings than White women. Factors such as stigma, challenges in accessing care, inadequate health insurance, mistrust of healthcare practitioners, and limited health literacy continue to pose substantial obstacles for many in this demographic when trying to receive mental health services (Okeke, 2013). Though uplifting, cultural tropes such as the “Strong Black Woman” and “Superwoman Schemas” can also impede the use of mental health services by encouraging unhealthy coping mechanisms (Watson and Hunter, 2016; Woods-Giscombé, 2010; Watson and Hunter, 2015; Okeke, 2013). The discrepancies in the use of mental health services are especially concerning because untreated depression has serious consequences that include a lowered quality of life, decreased productivity, and poorer health outcomes (Kessler and Bromet, 2013; Chen et al., 2005).

Understanding the state of mental health among Black women necessitates delving deeply into the complex web of their lived experiences in addition to their sociocultural background. There exist two diametrically opposed viewpoints of Black women in America (Allen and Britt, 1983). The first emphasizes inequalities due to less access to economic opportunities, racism, and sexism, portraying Black women as the most disadvantaged group when compared to middle-class White men (Allen and Britt, 1983; Pinderhughes, 1989). According to this viewpoint, Black women are more vulnerable to negative social and psychological experiences because of their intersectional status at the bottom of societal hierarchism (Belle, 1990). On the other hand, a different recognized perspective is the Black women's tenacity, fortitude, and flexibility, recognizing their abilities to work through and overcome structural obstacles to support their families, persevere through adversity, and forge their own paths to empowerment and self-actualization (Stack, 1997). Together, these differing perspectives provide a nuanced picture of the Black woman's experience of race, gender and class in America, highlighting the complexity of their existence and how it is affected by the intersections. Black women experience a particular kind of oppression that cannot be fully comprehended via the lenses of sexism or racism alone, despite experiencing a confluence of difficulties rooted in their intersectional identities (Brown and Keith, 2003; Belle, 1982; Brown et al., 2016).

This paper explores pivotal moments in mental health support for Black women, marked by an increasing array of resources with diminishing stigma. As options expand from traditional providers such as psychiatrists, psychologists, and social workers to tech-enabled platforms like social media and podcasts, Black women's engagement with these tools offers a window into the shifting paradigms of mental health care. Prominent resources such as Therapy for Black Girls^®^ (by Dr. Joy Harden) and the Homecoming™ podcast (by Dr. Thema Bryant) illustrate these trends, while public discussions by celebrities like Simone Biles, Megan Thee Stallion, Taraji P. Henson, and Regina King further signify a broader cultural shift toward openness in addressing mental health challenges. However, the use of mental health services remains low for Black women. The purpose of this study was to understand the attitudes and perceptions of Black American women toward using mental health services. The following research questions were explored: (1) What are the attitudes and perceptions of Black American women toward using mental health services? and (2) What are the barriers to using mental health services?

## Methods

### Procedures

Focus groups were conducted with women that identified as Black. Participants were recruited via the *Attitudes Toward Seeking Mental Health Services and Use of Mobile Technology Survey* (McCall, 2020) (i.e., survey respondents indicated if they would like to be contacted about an opportunity to participate in the focus group), posts on social media (e.g., Twitter), and flyers posted in the Durham and Chapel Hill, North Carolina communities inviting women (18 years or older) who identified as Black or African American or multiracial (i.e., Black or African American and another race) and had a history of anxiety or depression to participate in the study. However, study participation did not require a clinical diagnosis of anxiety or depressive disorder. Participants received a screening call to assess eligibility. Results from a study by Guest and colleagues revealed that more than 80% of all themes are discoverable within two to three focus groups, and 90% of themes could be discovered within three to six focus groups (Guest et al., 2017). Therefore, four focus group sessions were conducted. Each session was capped at five participants to allow for all participants to fully engage in the discussions.

In January 2020, the focus group sessions (5 participants each session) were held in Durham, NC in a private meeting room at a county library and at the University of North Carolina at Chapel Hill. TM (health informatics and mental health disparities researcher) moderated each session, and a notetaker was present to take notes on body language, non-verbal cues, and emerging themes. The moderator met with the notetakers to discuss non-verbal cues to note (e.g., hesitation to speak). Additionally, one of the notetakers was trained through a qualitative data collection course via the Odum Institute for Research in Social Science at UNC, and through previous notetaking while conducting interviews and focus groups with Black and Latinx women as a graduate research assistant. Before the sessions started, the moderator went through consent forms with participants and obtained their signatures. In addition, participants were informed that the study would last approximately 1 h to 1 h 15 min. Participants were advised that the session is confidential and that anything shared in the room should not be discussed outside of the session. The sessions were recorded using an audio recorder. Each participant received a $25 gift card and an information sheet with mental health resources (e.g., therapist directory, Therapy for Black Girls^®^ podcast) to use personally or share with others. The moderator and notetaker debriefed after each session. All participants were given a study participant ID (PID). The transcripts were de-identified, with all possible identifiers removed and names replaced with PID. All data was securely stored digitally (HIPAA compliant storage) or physical locations (locked file drawer and office). The study was exempted from full review by the University of North Carolina at Chapel Hill (UNC) Institutional Review Board (IRB # 19-2548).

### Measures

A focus group interview guide was developed to help the moderator facilitate the discussions (Appendix A). The interview guide was developed in consultation with a qualitative data expert at Odum Institute for Research in Social Science at UNC to better tailor the questions and improve the flow of the discussion. Characteristics of study participants, such as age, race, and education were collected during the screening call to assess eligibility. The focus of this paper is on the first part of the focus group sessions where participants were asked questions about the following topics: (a) past and current causes of stress, anxiety and/or depression, and coping skills used; (b) their attitudes and perceptions toward mental health and receiving mental health treatment; and (c) times in their life they felt anxious or depressed, and what type of support and/or resources would have been helpful to have access to.

### Data processing and analysis

The waveform audio files (.wav) were professionally transcribed and imported into NVivo 12 software for analysis (Ltd QSRIP, 2018). TM (focus group moderator) compared the transcripts to the audio files to confirm accuracy. TM and a licensed clinical mental health counselor served on the analytic team, and independently coded the first interview using a grounded theory approach to inductively produce an initial list of the emerging topics from focus group discussions. A consensus was reached between the coders on the reoccurring topics. The remaining three focus group interview transcripts were coded independently using the agreed upon themes/subthemes, then the analytic team convened to discuss their analyses and explore the data through questions and comparisons of all four interview transcripts for additional themes/subthemes. The coding procedures facilitated a collaborative analytic process (Saldaña, 2021).

Descriptive qualitative content analysis was conducted for all focus group transcripts. “Qualitative content analysis is a dynamic form of analysis of verbal and visual data that is oriented toward summarizing the informational contents of that data” (Sandelowski, 2000). Furthermore, a pragmatic qualitative research approach was employed to offer a “comprehensive summary of events in the everyday terms of those events” (Sandelowski, 2000) for responses to questions that describe personal experiences or opinions. For example, a participant may describe past experiences using mental health apps and events that caused them to be concerned with using the apps. In February 2024, additional secondary data analysis of the transcripts was conducted to further explore previously identified themes and note new themes the coauthors discovered (TM, MF, HT, and BA). Thematic saturation was reached, as there was no new useful information produced after analysis of all transcripts.

## Results

**Figure 1:**
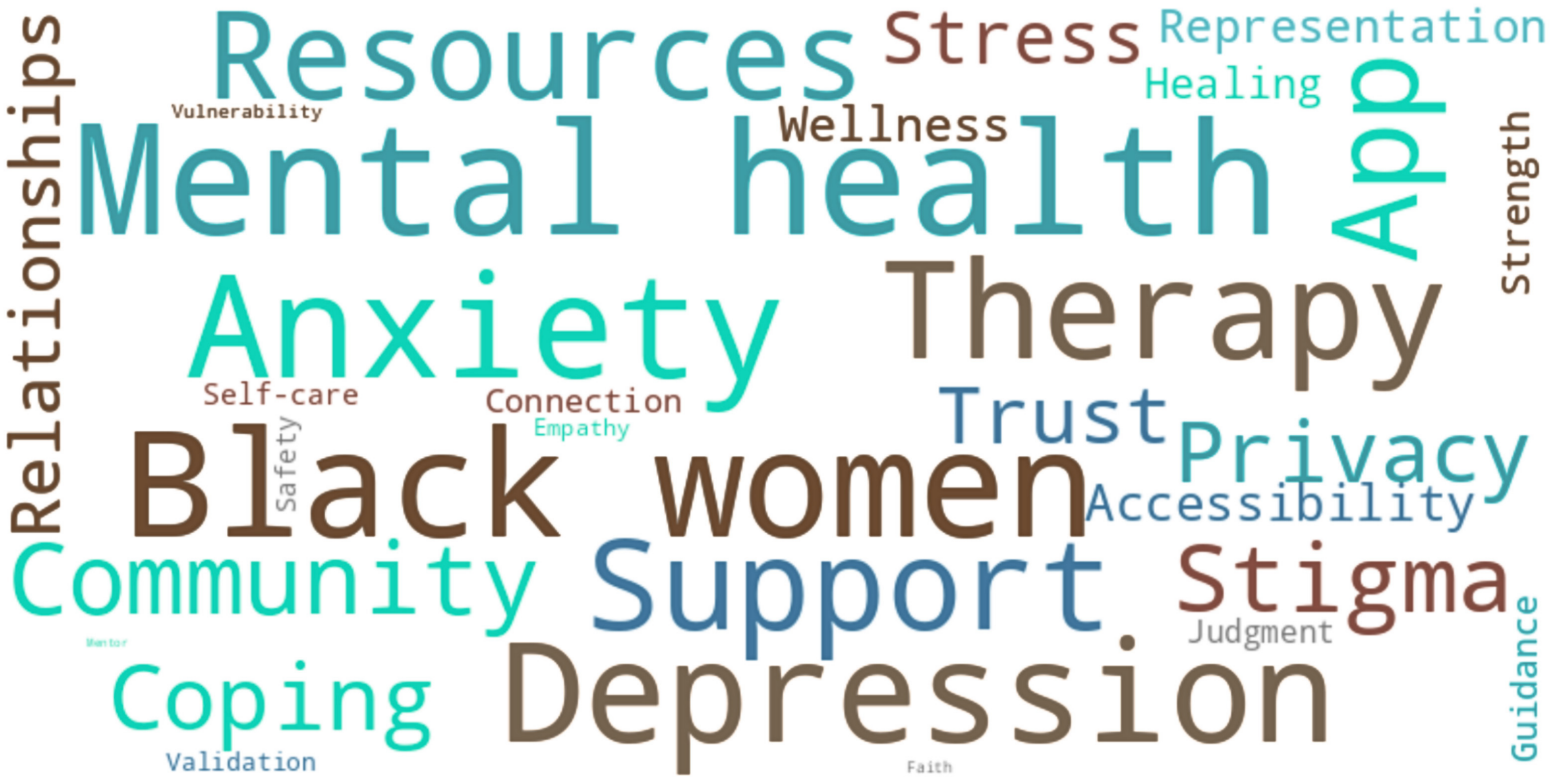
Word cloud of the most common words used in discussion of Black women's mental wellness.

Twenty participants (Sandelowski, 2000) attended the focus group sessions. Each focus group consisted of five participants. Participants could only attend one focus group session (approximately 1 h to 1 h and 15 min). Study participants ranged in age from 21 to 79 years (mean age of 36.6 ± SD 17.8 years), and all identified as female and either Black/African American or multiracial. Most participants obtained a bachelor's degree or higher (19/20, 95%). To engage a wide range of women and facilitate intergenerational discussion, half of the focus groups were mixed to include at least two participants aged 50 years or older. For comparison, one focus group consisted of women under the age of 30 years old. Participants' responses were summarized by the most prevalent themes (Appendix B), and a word cloud was created to visualize the most common words from the discussions and participants' sentiments (Figure 1).

### Themes

#### Defining mental health

In general, participants shared similar thoughts on what defines good mental health. Good mental health was primarily viewed as being aware of one's triggers and emotions, and having the ability to control one's emotions and express how you feel. Focus groups 1 and 3 emphasized self-awareness. Focus groups 1, 3, and 4 acknowledged the importance of having good coping techniques or the ability to express how you feel. However, participants in focus group 2 voiced that good mental health was the absence of stress and negative emotions:

“No stress.” [Speaker 3, Group 2, 29 years old]

“Laughter, joy.” [Speaker 1, Group 2, 27 years old]

Bad mental health was manifested both internally (e.g., negative mood, avoidance of dealing with issues) and externally (e.g., using food or alcohol to cope). Group 2 focused on internal manifestations of bad mental health. For example, a participant stated that bad mental health is:

“A consistent negative mood.” [Speaker 5, Group 2, 25 years old]

Focus groups 1 and 3 acknowledged that avoiding dealing with your feelings, issues, or situations are signs of bad mental health. One participant stated the following regarding avoidance and coping:

“I think of like bad mental health as avoiding the things, but trying to compensate them with other things. So like for instance…eating or drinking or like just doing things to kind of make you feel like you've escaped but you haven't really actually dealt with the issues.” [Speaker 4, Group 3, 29 years old]

Participants in groups 1 and 4 voiced that lack of resilience contributes to bad mental health.

“Lack of resilience. So, normal kind of run-of-the-mill things can break you down that shouldn't.” [Speaker 2, Group 1, 36 years old]

In general, positivity, resilience, self-awareness, and knowing how to cope with stressors was seen as indicators of good mental health for Black women. Avoidance was mentioned as a sign of bad mental health, along with using food and alcohol to soothe instead of addressing issues.

#### Black + Woman

The participants were vocal about the things that Black women deal with that other groups might not have to deal with. The intersectionality of being Black and a woman was discussed by all groups. One participant summarized their experience in stating:

“…I always said that Black women are hypervisible but also overlooked at the same time. So, it's just like people are like you're the only minority in the room. People are just looking at you for all your reactions and stuff. So any little thing you do is just exasperating and like, “Oh, you're dramatic.” Or, “You're too woke,” or something. And they just overlook everything that's very valid about what [you] say.” [Speaker 5, Group 1, 23 years old]

Participants voice that external stressors (e.g., racism, sexism, and caregiver responsibilities) resulted in negative emotions that are deleterious to one's mental health (e.g., loneliness, increased stress, and feeling of constantly being policed). The compounding negative effects of having to continuously deal with microaggressions due to racism and sexism were discussed. For example, one participant stated the following about the workplace:

“We're always at the fringe. And so even I find it like as I moved up in my career in my first director position, it was me at the table really, even amongst women, right? Not that there are many women. And so that was the first time I think I was able to get away from just racism. It was like, ‘Oh, this is like a whole thing. Y'all are saying that, microaggressing me race-wise, but also because I'm a woman. This is weird. What is happening here?' Whereas generally we're in more homogeneous groups, we're getting one or the other, so it's not so bad. But when we're in situations where we're just, where everything's compounded, it's like, ‘Whoa, who can I go to?' Which is why I think we end up leaning on each other much more than other folks.” [Speaker 2, Group 1, 36 years old]

Focus groups 2 and 3 expressed that Black women are constantly policed on everything including the tenor of their voice and are often faced with navigating stereotypes that cast them as aggressive and hard to approach. As a result, Black women are often stigmatized and treated as socially impalpable, leading to isolation, misunderstanding, and a persistent sense of being undervalued. A participant in focus group 2 provided an example of how being perceived as aggressive impacted her school life:

“Case in point last semester…my end of term evaluation, and there was only one other Black woman in the room who was a professor I have not had, because she doesn't deal with the first year graduate students, and multiple times in my feedback [that] was given, the word ‘aggressive' was used. I had [the] teacher say, ‘There were some times I wasn't sure if I should give you a note, because I wasn't clear on how it was going to be received, or if there was going to be some resistance,' and I'm thinking that's not my problem, and second of all you're the teacher.” [Speaker 3, Group 2, 29 years old]

“After that I spoke to the Black faculty member. [They] emailed me and was like, ‘Hey, how're you feeling?' Afterwards, we had a conversation about it, and she was just like, ‘I just want you to know that afterwards I emailed everyone in that room about how that language is inappropriate, and how historically has been used to label Black women if they're passionate, if they're excited, if they are upset. It doesn't matter. If they're anything except neutral - then they're aggressive'.” [Speaker 3, Group 2, 29 years old]

Regarding, navigating “white spaces” (i.e., places where White people are the majority) one participant voiced:

“The constant pressure of just what it means to be in those ‘white spaces,' and feel like you're in the spotlight to speak for your own group or to make yourself present, because you can quickly be swept aside if you're not vocal, but then that slippery slope if you're too vocal now you're the angry Black woman, instead of being passionate about what you feel.” [Speaker 4, Group 2, 23 years old]

Other participants noted their experiences regarding their role in the overall Black family dynamic, including being a single parent and serving as the “pillar of strength.”

“I think there's a heavier burden on Black women with their families. I feel like sometimes like, you know, like you don't always have the luxury of being able to like stay at home or like when you work then you actually have to go home, you have to cook, you have to do all of these things. Like, you know, you don't have a lot of assistance and everyone is looking for you to be that person and for you to always be the strong person in the house. Like mom can't cry. Like what's wrong? Like, you know, and I think you have to not only have that persona at work, but also have that persona at home too.” [Speaker 4, Group 3, 29 years old]

Participants in groups 1 and 4 brought up the point that Black women historically have not prioritized maintaining good mental health. Instead, the role of caregiver has been the priority and mental health an afterthought:

“Some days [with] taking care of everybody you really don't have time to take care of yourself.” [Speaker 4, Group 4, 62 years old]

One participant expressed how their experiences influence their decision regarding having children.

“Moms in general stress about their kids being safe, but in the last 5 to 6 years I've literally been like, ‘You know what? I don't think I'm going to have kids, because regardless of who I procreate with they'll be brown. They'll be considered brown,' and not only do I have to worry about just the day to day don't get hit by a car, or do we have a stalker neighbor? But this person might feel uncomfortable around you and might just kill you. I mean Tamir Rice, that could have been my brother.” [Speaker 3, Group 2, 29 years old]

Overall, the participants felt that Black women were not given the same consideration or grace as their White counterparts. In addition to navigating the pressures of hypervisibility and caregiver responsibilities, fear of being labeled as angry, antisocial, too sensitive, etc. were highlighted as stigmas at the intersection of being Black and a woman.

### Factors contributing to anxiety and depression

Most of the factors that contributed to anxiety and depression were school or work-related (e.g., imposter syndrome and performance expectations), however the women also spoke about stressors in their home life. Some participants also express not having full understanding of factors that contribute to anxiety and depression.

“I saw this presentation on something called allostatic load, which is just all the small little racial comments and microaggressions that we go through. It adds just an emotional psychological load on you that you don't even realize you're carrying. And that's been building since you were born. You've been a Black woman your whole life. So, there's a lot of research about how all this small stuff affects people that they don't even realize.” [Speaker 5, Group 1, 23 years old]

Managing expectations of family members and caregiver responsibilities were stated as causes of anxiety. Specifically, parenting and conversely dealing with expectations from one's parents were both identified as being anxiety inducing. One participant shared:

“My anxiety definitely comes from being the first person in my family to do a lot of things. And like you were saying the pressure is immense but it's more of like pressure I put on myself because I have a younger sister so it's like whatever I do I want to do it great, so that she has someone to look at. And then my parents are like pressuring me cause they're like, we want you to be better than us. But it's, it's a lot for like just one person to maintain.” [Speaker 1, Group 3, 21 years old]

Some participants attributed their experience with anxiety in part to perfectionist ideas surrounding how they show up in the world, given the inherent responsibility of representation and hypervisibility. Another participant shared how hypervisibility triggered imposter syndrome in school settings. She expressed:

“And I guess my experience with anxiety, I'll just, I guess, lump it into imposter syndrome, especially with like in undergrad. I feel like I was anxious just the whole time because of the idea of existing in spaces where nobody looks like you, you're hypervisible but people aren't paying attention to you and you feel like you don't belong. So, it's just like, what do they call it? Double consciousness or whatever.” [Speaker 5, Group 1, 23 years old]

In focus groups 3 and 4, work-related stress was one of the primary causes of depression. One participant recounted an experience where anxiety was brought on due to having to push for an adequate raise for herself:

“I remember having conversation, I'm with one of my partner companies that I've worked for and as the chief financial officer at the time, he's going over the raises, and who he's giving raises too. And we're sitting at a table and he tells me what he's offering me and I kind of look up and I said, well that's not good enough and I continue to do my work. And at the same time I was so frightened about what he was going to say, but if I hadn't said it, my truth, knowing what I was worth and looking at what he was giving everybody else, you know, so he returned and he said, ‘Okay, I'll give you a ten thousand dollar raise.' We have to know our worth, [because] people perceive [us] as not being as worthy as they are. And that causes anxiety. People perceive [us] as not being as worthy as they are. And that's stressful.” [Speaker 2, Group 3, 61 years old]

Groups 1 and 3 discussed that financial stressors can cause depression. The harmful effects of these compounding stressors were summarized by another participant:

“I think all the stresses on top of each other because if there's just one thing you're worried about, then it's okay. But like when you're worried about your family, and finals, you're worried about finances all at the same time, that's like very overwhelming.” [Speaker 4, Group 1, 22 years old]

Compounding factors such as performance expectations from work and school, caregiving responsibilities, financial stressors, and continuous microaggressions were voiced as contributors to anxiety and depression among the participants. The participants were in consensus regarding feeling pressured to both represent your community in majority “white spaces” while feeling ignored and undervalued, such that you must advocate for what you deserve (e.g., a fair raise).

### Mental health maintenance and protective factors

The strength of Black women was a recurring topic among the groups. In general, participants voiced that the strength is gained by personal experience as well as being taught how to cope. One participant stated:

“…we raise our daughters, we love ourselves, we raise our daughters. I feel like Black women are raised, right? As in we are literally, this is what this is, this is what you might face, and when this happens you need to do this. And so I feel we have a lot of those thoughts kind of going daily.” [Speaker 2, Group 1, 36 years old]

In focus group 4, when asked, “what, if anything, do Black women do to maintain good mental health?,” participants attributed their ability to sustain good mental health with inherent strength taught to generations of Black women of which has both its benefits and negative effects:

“I don't necessarily know if there's anything different because I don't think I'd been taught how to cope well. So, I don't know. Maybe my experience is different. I don't know if there's something just inherently stronger about Black women or that we've been taught to suppress.” [Speaker 3, Group 4, 29 years old]

“I think going off of that, maybe not so much consciously or explicitly, but just observing how our mothers or other Black women in our lives have dealt with things and it's just kind of powering through and I think to an extent having that modeled can be helpful, but at the same time it has that, that has its shortcomings as well when sometimes you really just can't just keep going. It's that bad. You do need some additional support.” [Speaker 2, Group 4, 23 years old]

The focus group participants shared that talking to their girlfriends is one of the most common ways that Black women maintain good mental health. For example, one participant stated:

“No, seriously, I think historically and to some degree having a girls' night out, or having at least one girlfriend to kinda talk to and decompress with helps, even if they're not saying nothing back. Just to know that somebody's listening.” [Speaker 3, Group 2, 29 years old]

Unanimously resiliency was stated as the top quality (i.e., protective factor) Black women have to help them to maintain good mental health. However, participants in focus groups 1 and 2 discussed the duality of resiliency, it can be both helpful and harmful. One participant stated:

“I also say resilience, though that could quickly become a double-edged sword or be a really tight rope to walk on, but I think Black people in general, Black women especially, you have to be resilient in order to survive all that previous generations have had to overcome and be put through, so I think it is built into our very foundation, resilience. But I think the idea of strong Black woman has also hampered us from seeking solace and using other resources to better our mental health or to talk about our mental health in general, so I think it's a fine line, but I think resilience fundamentally has helped Black women be strong.” [Speaker 4, Group 2, 23 years old]

The participants felt that Black women were taught how to be strong because they had to be—taught by the Black women in their lives through verbal instruction and observation. Lessons on how to navigate this world as a Black woman has been passed down through generations. However, the same protective factor, “resilience,” has also taught them how to suppress which negatively affects one's mental health.

### Coping and support

Discussion about coping behaviors revealed that the participants actively seek constructive outlets to help them deal with the things that caused them to be anxious or depressed. These activities include spending quality time with others, practicing hobbies, and introspection. For example, one participant stated:

“…I've learned to pick up hobbies, like distractions or things that I know would calm me.” [Speaker 1, Group 1, 29 years old]

Participants also disclosed that food and alcohol have helped them to cope in the past, whether it was eating or having a glass of wine alone or with a group to decompress. Overall, participants were open about the coping behaviors they felt were helpful, However, groups 1 and 4 discussed that avoidance is used as a coping technique, and that ignoring issues can be harmful. A participant voiced:

“…I kind of just ignore [the issue]. I'm really good at compartmentalizing and acting like I don't feel a certain way, which isn't good.” [Speaker 5, Group 1, 29 years old]

Regarding support for managing anxiety and depression, the majority of participants voiced that it would be helpful to have someone to talk to when they are anxious or depressed. Participants in groups 3 and 4 spoke positively about informal sources of support such as friends or mentors. Conversely, some participants expressed feelings of isolation as they struggle to find someone who understood what they were dealing with, be it among family, friends, or professional support resources.

“I feel like I'm by myself or I don't like, I can't talk to my parents or my brother about what I'm going through because they haven't been where I'm at. And so for me, good mental health would be just being able to speak to someone who can relate to what I'm going through. So I don't have to try to explain and have someone like sympathize but not empathize.” [Speaker 1, Group 3, 21 years old]

Focus groups 1 and 2 expressed the need for professional sources for support (e.g., therapy, campus health services, and resources). For example, one participant shared:

“When I was experiencing, like I mentioned, burnout from school and just feeling really pressured from that, I did seek treatment with a Licensed Clinical Social Worker and that was really helpful. It wasn't through my school, but it was just someone who had a private practice near where I lived and I found that to be really helpful.” [Speaker 2, Group 4, 23 years old]

Similarly, another participant shared how helpful it was to seek professional support, stating:

“When I was working, at least twice I had a situation with my job and I actually did use the EAP program and I thought it was helpful because I knew I felt so much better. I probably went maybe once and it was just having somebody to talk to and they had a completely different perspective because they didn't know you. And I felt so much better and I was able to then deal with the situation that I was facing, in a positive light.” [Speaker 1, Group 4, 66 years old]

However, some participants recounted instances where they left feeling undertreated or misunderstood due to lack of cultural competency and empathy.

“I've literally had people telling me, ‘Oh, you should be way more messed up than you are.' And I'm like what is that supposed to mean? Just because I can articulate what I'm feeling better than most, suddenly that means I don't need help. I don't see what that means. But I think basically the cultural competence is the biggest part because there's tons of resources. I don't think there's a lack. I think it's just a lack of people looking like us in those resources. I was trying to explain, I guess how I had known I had been experiencing anxiety and depression, pretty much since I could remember. But I didn't say anything because Black families don't believe in that. And she's like, ‘Oh, what do you mean by that? What do you mean that people don't believe in mental health in the Black community?' And it's just realizing how much you'd have to catch them up on stuff just to get them to understand where you're coming from. And just the way they look at you like a specimen. It's weird and I don't like it.” [Speaker 5, Group 1, 23 years old]

Participants discussed the use of medication for support with depression and anxiety.

“I'm very concerned about medication being part of the conversation or introduced. Unless I introduce it. And I don't know if this is part of my hypersensitivity, just working on college campuses too, but I feel like it's such a prevalent part of the conversation now. I just, I don't want to have that conversation and I feel like my experience in the past with therapists has gone there a little faster than I was comfortable with. I was like, I want to try, there has to be other things that I can do too. Right? Short of being diagnosed with something where it's like I'm not concerned if I'm diagnosed with something. Like we've gone through this and you feel like, you're going to need this. But I feel like the medicine conversation, for me, feels like a barrier too. And I would probably think for a lot of Black people.” [Speaker 2, Group 1, 36 years old]

Another participant added:

“You need to listen to me first before you try to give medication to me.” [Speaker 5, Group 1, 23 years old]

Participants in focus group 1 also discussed the use of apps to support their mental health (e.g., meditation and breathing apps), in addition to podcasts tailored to Black women audiences. One participant shared:

“[T]here's a lot of podcasts that I feel like geared toward African American women, like Therapy for Black Girls that's like one of my favorite ones. I think there's another one called the Balanced Black Girl and Sarah Roberts, TD Jakes daughter, has a really good podcast.” [Speaker 4, Group 3, 29 years old]

In response another participant stated the need for more culturally competent apps:

“I was going to say that but I guess my problem with the apps, maybe [the need for] more culturally competent meditation apps and stuff like that.” [Speaker 5, Group 1, 23 years old]

The sentiment that there is a need for more culturally competent mental health apps was also echoed later in the focus group discussions.

### Attitudes toward using mental health services

Most of the participants endorsed using mental health services, such as seeing a therapist.

“I love therapy now, but I think it was uncomfortable in the beginning, because it was like telling your business to somebody, to a stranger, and I was raised in a way you don't tell your business to people. You get in trouble when you're little and go to school and you just tell your teacher everything, so it was really hard to unlearn that, so me and my therapist would just sit and look at each other. She'd ask me a question and I'd give her really short answers, because I was like, ‘This isn't… This doesn't feel good. This isn't normal,' so I think that there's a stigma with therapy, but I think also with the way that we handle things. We're in the mode of like, ‘Okay, you take care of it yourself,' and so it's really hard to put that on somebody else who you don't know.” [Speaker 1, Group 2, 27 years old]

Every group expressed the preference for a Black female therapist. The emphasis on having someone that looks like them was due to bad experiences in the past, or the perceived burden of having to explain to the therapist what Black women, in general, deal with. Lack of cultural competency, mistrust, and lack of empathy were the main reasons for not initially seeing a therapist or discontinuing treatment. Regarding preference for a Black female therapist, one participant stated:

“I think it's excellent. If you can find somebody, I personally feel they have to look like me and know where I'm from. No disrespect to others. You have to know the person that you're getting ready to sit down and talk to and understand the culture and the background and the stigma to make them feel comfortable enough to open up. And I feel then we would open up to say yes to medication and because I think that's a healthy part of trying to relax the body and calm the brain and get you to focus…I don't feel that it would be a problem as long as everything is managed properly. So I'm in for seeing a therapist.” [Speaker 2, Group 3, 61 years old]

Focus group participants also identified cost, lack of time, and lack of transportation as additional barriers to seeking treatment.

“I think being on a college campus makes it easier to have resources. I think people are making a big deal about mental health now. I might be wrong but I'm in the public health school, so mental health is one of the departments, but I think outside of college campuses, it makes it very hard because it's not affordable to people or close or accessible or any of those things.” [Speaker 4, Group 1, 22 years old]

Another participant added how apps have made accessing resources easier compared to previous years:

“The nice thing is that we do have apps now because I'm thinking about, I used to live in a rural area. I always had to drive out of the area to go to a doctor and there's telemedicine nowadays and then you can use your app for therapy or coping mechanisms. So, that's a big step because I'm pretty sure like 10 years ago I probably wouldn't have been able to find a therapist in that area.” [Speaker 1, Group 1, 29 years old]

A generational difference was observed in the attitude toward using social media as a tool to maintain good mental health. The younger focus group participants were more likely to use social media as an outlet and a tool by expressing how they felt, connecting with others, and finding mental wellness resources (e.g., information about podcasts, encouraging or informative tweets). Whereas, the older participants were less likely to use social media or deemed it inappropriate to use as an outlet to express oneself. For example, one of the older participants stated:

“I'm not into all that social media stuff. So, I remember one time…Valentine's Day, [my daughter] called me her date and she had to tweet that, ‘Having Valentine's Day with my mother.' And I don't know if that got something off her chest because she didn't have a boyfriend or what, but I just didn't understand. Why'd you tell everybody that? Who cares? So, I'm not into the social media thing. So, I don't know about people that are into social media, I guess, we sometimes think that what the young people put out there is not appropriate.” [Speaker 3, Group 1, 63 years old]

In response, younger focus group members expressed that using social media can be helpful for maintaining good mental health. One participant stated:

“I will say that my perception of social media and mental health, I think it's helped a lot because it gives a lot of people access to other people's thoughts and feelings and understanding like, ‘Oh, I'm not the only one experiencing this.' Or people who have resources already for people who don't have access to them elsewhere, that's a good place for them to access it.” [Speaker 5, Group 1, 23 years old]

Everyone agreed that there is still stigma in the Black community around using mental health services. Needing mental health care is seen as a weakness or lack of faith, and people are afraid of being judged and labeled as “crazy.” Participants discussed the relationship between the church and mental health support, expressing the importance of faith to their community while also emphasizing the influence that church leaders have on their members' attitudes toward seeking mental health services.

“We need to become more spiritual, go to church, yes, and learn the bible, but we really need to be able to speak one on one and get what is going on within our mind. What chemical imbalances there are, that's going on because that's what, you know basically what I feel that it is. Your pastor can't tell you that. He studied and went to school for theological seminary. But he did not go to medical school to understand the brain and how the brain functions and the behavioral science. So I fault a lot of seniors in church who follow that. You have to understand, that's not what his calling was, to diagnose my issues. He is to teach me how to pray to God if that's what I want to do. So I think those walls have to come down for the Black community.” [Speaker 2, Group 3, 61 years old]

Although there is still stigma around using mental health services, all of the participants agreed that the stigma has decreased in the past 5 years. However, the sentiment was that there is still work to be done to normalize therapy. Regarding the stigma around using mental health services changing in the last 5 years, one participant stated:

“I think it has [improved], but it's still some work need to be done. More people are talking about it now than they used to.” [Speaker 4, Group 4, 62 years old]

Focus groups 1 and 3 acknowledged that subgroups within the Black community (e.g., young adults and college graduates) have less stigma around mental health than others, and are acting as catalysts for change. For example, a participant voiced:

“I think a lot of, especially our younger generation, a lot of us are like, that's what we study. That's what I went to go study. So we're going back and having these conversations with our family and informing them about their own mental health and they're seeing us, I guess, unearthing the things that we need to talk about and it like inspires them to do the same thing. I've seen that with my own family and I know with my friends that's kind of been the same thing. So, I think we're on a cusp of some change.” [Speaker 5, Group 1, 23 years old]

In order to really see the destigmatizing of mental illness in the Black community participants stated the need for more education on mental illness and available resources (e.g., therapy), and increase in social support to discuss mental health needs and seeking care. Specifically, participants mentioned that churches should work on messaging to normalize mental illness and seeking treatment.

## Discussion

### Principal findings

Past studies have shown that African American women's double-minority status contributes to increased stress (Greer, 2011; Thomas et al., 2008; Lewis et al., 2016; Newton, 2023; Miller, 2023). The intersectionality of being a double-minority, both Black and a woman, was discussed by all focus groups. The term “intersectionality” was coined by Crenshaw to explain how the “intersectional experience [of being both Black and a woman] is greater than the sum of racism and sexism, [and] any analysis that does not take intersectionality into account cannot sufficiently address the particular manner in which Black women are subordinated” (Crenshaw, 1989). Participants voiced that the compounding effects of having to continuously deal with overt racism and sexism, as well as microaggressions, resulted in feeling negative emotions that are harmful to mental health (e.g., feelings of loneliness, increased stress, and feeling constantly “policed”). African American women are “policed” on everything from hairstyles to how they expressed themselves and have to learn how to navigate “white spaces” (i.e., places where White people are the majority), causing feelings of inadequacy and stress (Robinette, 2019). Therefore, Black women have to create spaces that feel safe, which means a necessity for relationships with individuals that share their socio-cultural background as exemplified by the number of Black participants who prefer a Black woman mental health professional. Furthermore, the biopsychosocial consequences of stress from race, gender, socioeconomic status (SES), and age discrimination are supported by the literature such that Brown et al. (2016) showed racial-ethnic, gender, and SES stratification intersect, which underscores Crenshaw's original theory about intersectionality. In this study we used educational attainment as a proxy for SES; therefore, future studies would benefit from collecting these data. Also, that results differ by age is worth further investigation.

Our findings support previous work, including Newton (2023), which explored the oxymoronic co-occurrence of hypervisibility and invisibility experienced by Black women on predominately white campuses. This research denotes how microaggressions simultaneously acknowledge the physical presence of Black women while ignoring and invalidating their experiences (Newton, 2023). Jones (2020, 2022, 2023) demonstrated the silencing that Black women often face, with rare opportunity to directly confront their perpetrators or express their hurt and anger. The additional stressor of having to be cognizant of how they present themselves (e.g., suppressing emotions to avoid perpetuating harmful stereotypes such as the “angry Black women”), was noted as anxiety inducing, leading to a silencing effect (Kilgore, 2018).

Most of the participants endorsed using mental health services, such as seeing a therapist. However, every group expressed the preference for a Black female therapist. The emphasis on having someone that looks like them was due to negative experiences in the past, or the perceived burden of having to explain to the therapist what Black women, in general, deal with, including the importance of speaking the same “language.” Lack of cultural competency, mistrust, and lack of empathy were the main reasons for not initially seeing a therapist or discontinuing treatment. While some clients may have no preference, Black Americans who indicate higher levels of mistrust of White individuals are more likely to discontinue therapy before treatment goals are reached if they are seen by a White counselor (Terrell and Terrell, 1984).

Respondents identified many barriers to seeking treatment. Most of the barriers were related to cost, not knowing where to get services, lack of time, stigma, concern that they might be committed to a psychiatric hospital or might have to take medicine, difficulty finding a preferred provider (i.e., African American woman), and concerns about confidentiality (McCall, 2020). These barriers are consistent with those documented in the literature (Hines-Martin et al., 2003; Merritt-Davis and Keshavan, 2006; Thompson et al., 2004). Additionally, the findings were consistent with results from a previous study which surveyed Black American women regarding barriers to seeking care. Although survey respondents had favorable views toward seeking mental health services, approximately 40% of respondents indicated that during the past 12 months there was a time when they needed mental health treatment or counseling but didn't get it (McCall, 2020). This finding revealed that lack of awareness of personal need for mental health care may not be the primary reason why Black American women might not seek mental health care. The use of mobile technology may help to eliminate or mitigate some of these barriers to receiving mental health care (McCall et al., 2021, 2022).

Good mental health was primarily viewed as being aware of one's triggers and emotions, and having the ability to control one's emotions and express how you feel. Self-awareness and the importance of having good coping techniques was emphasized by the focus group participants. Conversely, bad mental health was manifested both internally (e.g., negative mood, avoidance of dealing with issues) and externally (e.g., using food or alcohol to cope). Lack of resilience was identified as a major contributor to bad mental health. Ward and Heidrich (2009) found that African American women's preferred coping strategies included praying, using informal support networks (e.g., friends), and seeking treatment. Our findings support this since the focus group participants agreed that talking to their girlfriends and seeking solace in religion were the two most common ways that Black Women maintain good mental health. However, the participants voiced that Black women historically have not prioritized maintaining good mental health. Instead, caregiving responsibilities has been the priority and mental health an afterthought. Additionally, participants emphasize the influence of outdated perspectives on mental health held by church leaders' and elders can deter people from seeking mental health support from licensed professionals. Participants did not discourage the use of spiritual practices, such as prayer, however they recognized the need for trained mental health professionals to provide care for individuals experiencing mental health challenges.

There was a generational difference observed in attitudes toward using social media as a tool to maintain good mental health. The younger focus group participants were more likely to use social media as an outlet and a tool by expressing how they felt, connecting with others, and finding mental wellness resources (e.g., information about podcasts, encouraging or informative tweets). Whereas the older participants were less likely to use social media or deemed it inappropriate to use as an outlet to express oneself. Younger African American women (< 50 years old) are more likely to use social media than their older counterparts (The Nielsen, 2017). Use of social media can foster a sense of greater social connectedness, feelings of belonging, normalize challenges and facilitate sharing of coping strategies by sharing personal stories about dealing with mental illness (Naslund et al., 2016).

Participants voiced that “resiliency” is the top protective factor Black women have to help them to maintain good mental health. Furthermore, the women espoused the idea of the Strong Black Woman/Superwoman role, a phenomenon that affects the experiences and stress reported by African American women. The Superwoman role is characterized by the “obligation to manifest strength, obligation to suppress emotions, resistance to being vulnerable or dependent, determination to succeed despite limited resources, and obligation to help others” (Woods-Giscombé, 2010). However, participants discussed the duality of resiliency in that it can be both helpful and harmful. One can depend on resiliency too much and delay seeking needed mental health care, which can have deleterious effects (Watson and Hunter, 2016), such as increased allostatic load caused by stress that can lead to cardiovascular and neurological consequences (Allen et al., 2019; Geronimus et al., 2006).

Although there is stigma in the Black community around using mental health services, all the participants agreed that the stigma has decreased in recent years. However, needing mental health care is still seen as a weakness or lack of faith, and people are afraid of being judged and labeled as “crazy.” Work remains to be done to normalize therapy in the Black community. Subgroups within the community (e.g., young adults and college graduates) that have less stigma around mental health than others and are acting as catalysts for change. Partnering with churches and community organizations to promote mental wellness and normalize seeking treatment will help to destigmatize mental illness with the Black community.

### Limitations

Participants in the focus groups shared common thoughts, recommendations, and opinions which are representative of their individual experiences; the results should not be generalized to all Black women. A limitation was that most of the focus group participants were under 50 years old, therefore, the sample skewed toward capturing the thoughts and opinions of younger Black women. This may limit the generalizability of the findings to older Black women. We did not collect income data to determine socioeconomic status; however, most participants obtained a bachelor's degree or higher—degree status can be used as a proxy for socioeconomic status. Moreover, due to the geographical restriction in recruiting participants, the results may not reflect the opinions and perceptions of a nationally representative sample. Additionally, given the stigma of mental illness in the Black community, participants may also have felt peer pressure to give socially desirable answers to the moderator's questions. These limitations can be mitigated in the future by adding one-on-one structured interviews with participants to reduce social pressure and potential “groupthink.”

### Conclusions and future directions

In general, Black women were more likely to seek culturally competent therapy for various mental health concerns, particularly anxiety and depression. This is promising because earlier studies have shown a resistance to mental health services among Black American communities. Black women face unique issues of marginalization revealed in this focus group and worth further exploration. Additional studies of mental wellness among Black women are warranted, particularly in the context of social determinants of health, to further understand the drivers (like socioeconomic status and lived environments) that lead to deleterious mental and physical consequences. In fact, understanding these drivers may provide a link between the mental and physical stressors that frequently lead to illness.

## Data Availability

The raw data supporting the conclusions of this article will be made available by the authors, without undue reservation.
