# Supplementary material for: “I think we're on a cusp of some change:” coping and support for mental wellness among Black American women
Source: Front Psychol. 2025 Jan 14;15:1469950. doi: 10.3389/fpsyg.2024.1469950 (PMC11772172; doi:10.3389/fpsyg.2024.1469950)
Supplement: Supplementary file 1 [file Data_Sheet_1.docx]

**Appendix A**

***Interview Guide Questions***

Let’s start the discussion by talking about how you define mental health.

1. When you think about “good mental health,” what goes through your mind?
2. When you think about “bad mental health,” what goes through your mind?
3. What, if anything, do Black women do to maintain good mental health?
4. Are there certain qualities that Black women have that help us maintain “good” mental health? If so, what are they?
5. Are there things that Black women deal with that other groups might not have to deal with?
   1. How do you think that affects our mental health? Can you talk more about that?
6. What are some of the things in the past that have caused you to feel anxious?
   1. How did you deal with them?
   2. What type of support or resources would have been helpful to have access to during that time?
7. What are some of the things in the past that have caused you to feel depressed?
   1. How did you deal with them?
   2. What type of support or resources would have been helpful to have access to during that time?
8. How do you feel about using mental health services, such as seeing a therapist?
   1. Do you think that there’s a stigma around using this kind of care? Can you tell me more about that?
   2. Do you feel that the stigma around using mental health services has changed in the last five years? Can you tell me more about that?
