# Supplementary material for: “I think we're on a cusp of some change:” coping and support for mental wellness among Black American women
Source: Front Psychol. 2025 Jan 14;15:1469950. doi: 10.3389/fpsyg.2024.1469950 (PMC11772172; doi:10.3389/fpsyg.2024.1469950)
Supplement: Supplementary file 2 [file Table_1.docx]

**Appendix B**

**Table 1. Participant responses by theme**

| Themes | Topics^a^ | Examples |
| --- | --- | --- |
| Black + Woman | - Racialized surveillance and microaggressions (10, 23, 24, 26) - Devaluation and invisible labor (9, 39) - The angry Black woman archetype (31, 40) - Assertiveness misread as aggression (22, 27) - Balancing kinship and caregiving (11, 12) - Hypervisibility and erasure (24, 26) - Generational expectations and survival strategies (21, 28) | “People who are supposed to be our allies and closely aligned with our struggles, from people appropriating our looks, and our features, and who we are, but don't want to be us and deal with the struggles that we go through, to existing in white spaces, to being able to count on one hand that you're the only Black woman in the room." *[Speaker 4, Group 2, 23 years old]* |
|  |  | “Walking into spaces that no one looks like you or I take a lot of, I guess, health and inequality classes. So, whenever there's discussions brought up about very sensitive things, I guess, or even if people are talking about politics and Trump, you have to cringe a little bit when someone makes a comment in class that's ignorant or something like that.” *[Speaker 4, Group 1, 22 years old]* |
|  |  | “Yeah. You have to mentally prepare yourself for that because anytime there's a conversation, you know somebody is about to say something and that ultimately is going to be harmful towards you but you're forced to gauge your reaction because you know it's not going to be perceived well.” *[Speaker 5, Group 1, 23 years old]* |
|  |  | “[S]ometimes depending on the store you go into, you may find that you're watched just because you're a Black person who walked in the store.” *[Speaker 1, Group 4, 66 years old]* |
|  |  | “In my department, for a long time, I was the only Black women, and I would be like, "Yeah, I really want to get braids, but then I'm going to have to reintroduce myself to every white person in the department, because they're not going to know that this Black girl who just walked by you is the one in your class," and it happened, and I was like ... It's a minor stressor, but it's still stress. It's still, “here we go”. I don't feel as important in this department, because you can't tell that the only Black person you interact with on Tuesdays and Thursdays changed their hair. Yeah, or even just the media attention around Black girls' hair. It's stressful, when you are watching it and thinking about that could be me, that could be my kids in the future. Things like that.” *[Speaker 1, Group 2, 27 years old]* |
|  |  | “[W]e just tend to have a more direct communication style than other populations, and so if we do decide to speak up or when we're pretty clear about stating what we need, you don't have to wonder or there's no message in the message, just whatever we say, is what it is.” |
|  |  | “And if you're matter of fact direct because you know, then you're angry or so it's, it's a new line that is, we walk every day where, you know, do I say something? You know, because I'm going to be judged. We're judged no matter what we say. It could be kind, it can be taken incorrectly. We could be persistent and then we're angry. Our tone and tenor is totally different from any other ethnic group and that can be a problem for a lot of people. So it's really difficult being Black, smart and we're just beautiful. It's hard for us and that's mentally draining.” *[Speaker 2, Group 3, 61 years old]* |
|  |  | “[A] lot of Black women are single parents. I think that takes a big toll on them because they have to do everything. They work and they have to take care of the child. They have to take care of themselves. And that can be taxing at times.” *[Speaker 4, Group 4, 62 years old]* |
| Defining mental health | - Awareness of triggers and proactive coping strategies (21, 40) - Developing effective coping mechanisms (11) | “Being aware of what triggers you and making provisions to either be away from those things or to have techniques to cope when they arise.” *[Speaker 2, Group 1, 36 years old]* |
|  |  | “I think it also comes down to being able to have good coping mechanisms for when you are faced with challenging situations.” *[Speaker 2, Group 4, 23 years old]* |
| Factors contributing to anxiety and depression | - Balancing kinship and caregiving (11, 12) - Hypervisibility and erasure (24, 26) - Generational expectations and survival strategies (21, 28) - The burden of representation (26, 39) - Imposter syndrome (28, 40) - Navigating white spaces (23, 28) - Strength and Resilience (9, 10, 39, 40) - Upbringing and family foundations (9, 10) | “I think there's mindset of perfection. Sometimes I think in the African American community costs a lot of anxiety. Like your hair has to be perfect, you have to look nice, like you know you have to, you know, do all the things that you're supposed to do. You know, like I have, for me, like I used to do my makeup for work in the mornings and now I just stopped because I'm like, I'm not waking up 30 minutes extra just to do my makeup. But like it's like when my hair being natural and I was like trying to make sure that it always looks halfway decent because I am the only Black person in my office. So you know what I mean? It's those things of like always trying to be like this perfect person because you're the representation of your group in this office and that can cause sometimes a lot of anxiety” *[Speaker 4, Group 3, 29 years old]* |
|  |  | “I think that, for me, in general, it's just how I was raised. Like you said, you're Black, so you're always going to stand out in a room. You've always got to be aware of how you look, how you dress, because you're always going to make an impression, because you're always going to stand out, and so for me I think that's caused some anxiety, like of how I grew up. Now I'm aware of who I'm around and how I dress, and what I say or what I don't say when I'm around other white people or just people in general, so I think for me that's my past.” *[Speaker 2, Group 2, 23 years old]* |
|  |  | "Our parents hold us to such a high expectation like they want us to do great and sometimes too, like I think that causes kids a lot of anxiety. Sometimes they feel that they can't always measure up to those expectations that mom and dad has. Cause they want you to be better than they were. And it's that constant like you have to do better, you have to do better." *[Speaker 4, Group 3, 29 years old]* |
|  |  | “[A] lot of Black women, in some degree, have the opportunity to experience an auntie or a big momma or a grandma that has exuded a level of unconditional love that's not normal for most people, and so even though you might not have the language to give to it, you've experienced it, and so I think sometimes we try to recreate that for other people, and what that turns into is sometimes not putting ourselves first, which can to your point easily become a slippery slope of now we're going down a bad road of mental health, because we're always putting other people before ourselves.” *[Speaker 3, Group 2, 29 years old]* |
|  |  | “For me, being in the health profession and working in a high-level stress job and dealing with having to take on other individual's issues plus balance [my own] and just not having that support of a, you know, supportive supervisor or a good working team that took a toll on me. And so I'm learning to navigate that and learning to say, you know what, I need some help.” *[Speaker 3, Group 3, 34 years old]* |
| Mental health maintenance and protective factors | - Upbringing and family foundations (9, 10) - Hobbies and leisure (13, 24) - Food, alcohol, and social bonding (27, 39) - Shared understanding and sister circles (23, 26) - Strength and Resilience (9, 10, 40) | “I think because we've been through so much, we're faced with a lot so it makes us stronger. We're able to handle adversity. We're used to adversity and having to handle it.” *[Speaker 1, Group 4, 66 years old]* |
|  |  | “And I think that's something that Black women have passed on. I almost feel like you teach your daughter, your niece, your granddaughter , whatever, you teach the next generation, how to deal with it or cope.” *[Speaker 5, Group 4, 28 years old]* |
|  |  | “[M]y college friends and I actually have been named our group chat "Sisters how you feel" and like that's just kind of how like we could talk about different things that we're all going through or just like funny stuff, gossip like on the blogs and like that. But I mean a lot of it is very like nurturing. Like if we were ever like a book, then we'll share this. Like, Oh, this book was really good. Like you guys should read it or like listen to this podcast or this TED talk and things like that. So I think just having a group of women who kind of support your mental health and you can feel like you can tell them when things are going wrong in your life and they're there to support you but not judge you, I think its a good like mental health for Black women.” *[Speaker 4, Group 3, 29 years old]* |
|  |  | “As I said, historically we've had to be very resilient people but I know that resilience in itself can create a loop of being too resilient, I guess. And taking more than you can handle or depending on that resiliency to get you through everything when you actually do need help from other resources.” *[Speaker 5, Group 1, 23 years old]* |
| Coping and support | - Hobbies and leisure (13, 24) - Food, alcohol, and social bonding (27, 39) - Shared understanding and sister circles (23, 26) - Isolation and lack of support (28, 40) - Suppressed emotions and avoidance (5, 21) - Assimilation and cultural negotiation (26, 27) - Seeking culturally competent care (6, 33) - Medication for depression and anxiety (36, 40) - Access and digital modalities (13, 38) | “I think shared lived experiences also helps because that builds the empathy between people when you talk about what's going on. So, it can be a better soundboard to what you're talking about.” [Speaker 5, Group 1, 23 years old] |
|  |  | “I think, for me, fortunately, I've had the…community, and that just provided so much joy, even though some of the topics we talked about were hard, but just to have that space to decompress with people, like this. It was low key group therapy, even though no one called it that. It was just like…we're about to go get some food and talk.” *[Speaker 3, Group 2, 29 years old]* |
|  |  | “If I don't have time to do one of my hobbies, I guess, if it's warm outside, I usually like just do whatever work I'm doing or plan for whatever's stressing me in the sun. During the winter time I guess it's a little bit harder.” *[Speaker 4, Group 1, 22 years old]* |
|  |  | “I agree with you, at work, if I am feeling anxious, I'll step outside and take a break.” *[Speaker 1, Group 1, 29 years old]* |
|  |  | “Wine. I usually, I mean, that's my thing to do with my friends. Wine Wednesday, just go and blow off steam for two, three hours.” *[Speaker 2, Group 1, 36 years old]* |
|  |  | “Sometimes you have friends and you call them and you say well look, such and such. [They say], “well you know you shouldn't have been there and you know you shouldn't have did that.” You might not want to hear that right now. You just want somebody that's going to say, “Hey, what's going on? Tell me about it” and not pass judgment. Just listen.” *[Speaker 4, Group 4, 62 years old]* |
|  |  | “I think having a space to talk about it, where it was known that it was a space to debrief…would have been helpful…Just thinking back to my younger years. Somebody naming what [anxiety and depression] were would be really helpful. It just seemed normal to be in class and so worried, and trying to be perfect and whatever sense, or feeling like blue, or something like that. If somebody had named it, and then I could have been like, "Okay, so there are support services for that?" *[Speaker 1, Group 2, 27 years old]* |
|  |  | “When I was experiencing, like I mentioned, burnout from school and just feeling really pressured from that, I did seek treatment with a Licensed Clinical Social Worker and that was really helpful. It wasn't through my school, but it was just someone who had a private practice near where I lived and I found that to be really helpful.” *[Speaker 2, Group 4, 23 years old]* |
|  |  | “I think I've had white, well non-Black therapists and a Black therapist and I feel like my experience with the non-Black therapists, well I've had two of them. One of them I only went for a couple of sessions because every time I talked to her she was really freaked out. I was just telling her what I feel is a normal life experience or something that I don't necessarily feel traumatized by and she's like, 'Oh no, poor you.' And it's just like, I just think a lot of times they lack the necessary empathy to be helpful." *[Speaker 4, Group 1, 22 years old]* |
|  |  | “And then in that same regard I had experienced that and then also I feel like this dismissal of Black women's pain, right, by default. I don't even necessarily know if they know what they're doing but I've had a therapist tell me before, it's like, you do your intake session. And he was like, "Oh my god. Your life has sucked." He couldn't even say I've had a life. In my little 30 something years. But he literally was like, "Well, I mean, no wonder you have some poor coping mechanisms but you're definitely not doing anywhere near as bad as you should given what you just shared with me." And it was like, "Well, that's a weird flex." Or I've had a therapist kind of tell me like, that was a white man. I've been to white women who failed to help me to make a plan. Right? My understanding of what therapy is, is that we want to have goals and things like that. And they just weren't doing that. I mean, I had one who kind of was like, "I don't really feel like you need to see me." I'm a doc candidate too. So she's like, "You're pretty lucid." Basically. "You know what's happening." I'm well spoken about my pain. I think they just thought, "Well, okay I don't know what to do with you." It's like you're not even trying.” *[Speaker 2, Group 1, 36 years old]* |
|  |  | “I feel like the medication conversation is weird because I'm technically prescribed, I just don't take it because I have my personal feelings over it. But I am trying, I guess, be reflective about it and realize that there's a spectrum to mental health and some people do need it but it's just whether or not I trust the person who gave it to me as to whether they really think I need it, or if they're just, like you said, just throwing it on you like, "Oh, here's some medicine, go deal with it." But not really thinking about, yeah this might help me, but I need coping mechanisms more. I need to talk about what I'm going through more before you throw it at me and after we go through all of it, if you still think I need it, then by all means I'll take it. My mom's on four different medications and stuff like that but she has severe mental health problems. So, that's why it makes sense for her but it doesn't make sense for everybody. But I feel like it's that nuanced conversation that doesn't have enough nuance when people present the idea to you.” *[Speaker 5, Group 1, 23 years old]* |
|  |  | “I'm like that. That comes from when you have children and they are quick to Black boys on ADHD medication and all that. I'm thinking, you're not listening to me but you're ready to give me some medicine. So no, I'm not going to take it.” *[Speaker 3, Group 1, 63 years old]* |
|  |  | “I know in undergrad my school had a Calm app subscription, that I didn't find out about until near graduation. But that's really helpful because they have meditations that range from five minutes to 30 or an hour or something. So, even if I only have five minutes, I was walking to my next class or something, I think that that helped to calm me sometimes.” *[Speaker 4, Group 1, 22 years old]* |
|  |  | “A lot of therapists or mental health workers have, I don't know about Twitter, but [have] Instagram accounts where they share videos or healthy coping mechanisms for certain things. And I know a lot of people find comfort in those types of things. I think they are more helpful than just stating a problem or something online.” *[Speaker 4, Group 1, 22 years old]* |
| Attitudes toward using mental health services | - Access and digital modalities (4, 13, 16, 38) - Revolution and systemic change (26, 39) - Mental health stigma and seeking support (4, 6, 16, 36) - Church and spiritual resilience (13) | “I think a lot of it can start in church too. Like having pastors who are like advocating for mental health. So like the church that I go to, the pastor is actually a psychologist. So I mean like it's so he's always pushing like mental health, you know, it's a totally different story than when people aren't, when the pastor or somebody who's closed minded to those too because then like they don't provide those avenues to their members. And I think a lot of it has to, because I felt like the Black community, we are so church like heavy I guess is the best word I can think about. But I think if we start, if it starts from the leadership that works it way down like, okay, these are services that I think can help you. And like, you know, it's okay to pray with people. It's okay to talk to them. But if you truly see that that person needs more than what you can provide, then like guide them into that avenue because they'll go, they'll be like, my pastor told me to come see you. They'll go like, but I think a lot of it has to start with them and then then eventually it will hit the rest of the body of the church. Just all being open minded to it." *[Speaker 4, Group 3, 29 years old]* |
|  |  | “That's so important. But you know, I honestly feel like, you know, to just talk about that further, I feel that that's the movement that's happening because there are so many openings of services. Which I feel serves everybody. So, you know, there was a service and church that I helped build with a young guy in New York city. The first thing they did was meditate before the service starts to get everybody in on one mind accord. You know, and breathing. We have to be open to what's going to help us move forward and not the traditional, the traditional doesn't work anymore. So I totally agree. It does start, it really, for us in the Black community, it's starts in that church. It really does.” *[Speaker 2, Group 3, 61 years old]* |
|  |  | “I think a lot of people who go to therapy, not a lot of people know that they're in therapy because it's like, “Oh well if they know that I'm seeing somebody then what are they going to think about me?” So I think a lot of it has to do with just opening up the idea of going to a therapist, like you go to your PCP and then once that happens I think a lot more people will utilize those services cause they're just such a huge stigma in our community about going to see a therapist. It's like, “Why are you going to see the shrink? Like you need to be praying.” You know, and it's just not open. I think a lot of people would love to use those resources but they fear the judgment that's going to come behind that with seeking therapy.” *[Speaker 4, Group 3, 29 years old]* |

^a^ These topics reflect key discussion points from focus group transcripts, with the denoted references offering context to those points.
